# Bone Microenvironment, Stem Cells, and Bone Tissue Regeneration

**DOI:** 10.1155/2017/1315243

**Published:** 2017-01-17

**Authors:** ZuFu Lu, Jenneke Kleine-Nulend, Bin Li

**Affiliations:** ^1^Biomaterials and Tissue Engineering Research Unit, School of AMME, University of Sydney, Sydney, NSW 2006, Australia; ^2^Department of Oral Cell Biology, ACTA, University of Amsterdam and VU Amsterdam, MOVE Research Institute Amsterdam, Amsterdam, Netherlands; ^3^Department of Orthopaedics, The First Affiliated Hospital, Orthopaedic Institute, Soochow University, 188 Shizi St., Suzhou, Jiangsu 215006, China

Despite the remarkable regenerative capacity of bone, the regeneration of large bone defects and the repair of nonunion bone fractures remain a major challenge in orthopaedic surgeries. Bone is the second most commonly transplanted tissue with over 1.5 million bone graft surgeries being performed annually in the United States [1]. However, the major limitations confronted with conventional bone grafts include limited availability and donor site morbidity for autografts and the risk of pathogen transmission for allografts [2, 3]. Given these limitations, there is a great need for developing novel and effective approaches for the regeneration of large bone defects and the repair of nonunion bone fracture.

Stem cell-based bone tissue engineering offers a promising approach for regenerating critical sized bone defects or repairing nonunion bone fracture. Understanding and recreating a signalling environment to control the differentiation of stem cells into the bone lineage would be of great importance. The components in bone microenvironment, which include a mineral phase (hydroxyapatite nanocrystals), an organic phase (composed of 90% collagen type I), a cellular phase (osteoblasts, osteoclasts, and osteocytes), and a soluble factor phase (growth factors and/or cytokines), provide a specific and balanced signalling network, which contribute to the innate bone metabolic and anabolic activities and maintain the structure and functions of the bone. Substantial efforts, therefore, have been made to mimic the bone tissue microenvironmental components for controlling the commitment of stem cells into osteogenic lineage cells for bone tissue regeneration. For example, by mimicking the bone nanostructure to engineering bone-related biomaterials, researchers have incorporated nanocrystals into biomaterials and demonstrated that they are effective in regulating various cellular functions including cell adhesion, proliferation, and differentiation [4, 5]; in addition, mimicking the signals provided by bone cellular phase (e.g., osteoblasts) has also been shown as a feasible approach to control stem cell fate into osteogenic lineage [5, 6]. Moreover, the cytokines and/or growth factors within bone microenvironment play a key role as well in the bone remodeling process, and mimicking their signals has been proven to be very successful in steering MSCs into bone lineage. Recently, inflammatory factors, transiently expressed by macrophages upon tissue injury, have increasingly been appreciated for their role in tissue repair and regeneration [7–9].

In this special issue, some cutting-edge original researches as well as review articles related to priming stem cell fate into the osteogenic lineage via mimicking the bone components (e.g., bone extracellular matrix, cells, and growth factors) were introduced and provided the readers with the updated knowledge and progression in the topic of bone microenvironment, stem cells, and bone tissue regeneration.

*ZuFu Lu* *Jenneke Kleine-Nulend* *Bin Li*
